# Successful treatment of metastatic bladder pleomorphic giant cell carcinoma with pembrolizumab

**DOI:** 10.1002/iju5.12281

**Published:** 2021-03-16

**Authors:** Kunihisa Nezu, Narihiko Kakoi, Fumiaki Tezuka, Shozo Ota

**Affiliations:** ^1^ Department of Urology Sendai Red Cross Hospital Sendai Miyagi Japan; ^2^ Department of Pathology Sendai Red Cross Hospital Sendai Miyagi Japan

**Keywords:** Bacillus Calmette–Guérin, bladder pleomorphic giant cell carcinoma, epithelial–mesenchymal transition, pembrolizumab

## Abstract

**Introduction:**

Bladder pleomorphic giant cell carcinoma is a rare and aggressive malignancy with a poor prognosis. There are no reports of immune checkpoint inhibitors for bladder pleomorphic giant cell carcinoma to date.

**Case presentation:**

A 72‐year‐old man presented with gross hematuria due to multiple bladder cancers. Despite transurethral bladder resection and intravesical injection of Bacillus Calmette–Guérin, bladder cancer recurred. Nineteen months later, he underwent total cystectomy. Pathological examination revealed bladder giant cell carcinoma. Twenty‐eight months later, pembrolizumab was administered due to para‐aortic lymph node metastasis. Forty‐four months later, the lymph node metastasis disappeared, and pembrolizumab administration was terminated. Fifty‐eight months later, the patient has remained in remission at the time of writing.

**Conclusion:**

Immune checkpoint inhibitors manifest a therapeutic potential in bladder pleomorphic giant cell carcinoma.

**Abbreviations & Acronyms**
BCG: Bacillus Calmette–Guérin
CD: cluster of differentiation
CECT: contrast‐enhanced computed tomography
CK: cytokeratin
ICI: immune checkpoint inhibitor
PD‐L1: programmed cell death‐ligand 1
TURBT: transurethral resection of the bladder tumor
UC: urothelial carcinoma
WHO: World Health Organization
β‐HCG: beta‐human chorionic gonadotropin

**Keynote message**

Bladder pleomorphic giant cell carcinoma is a rare and aggressive subtype of bladder cancer. This type of carcinoma is chemotherapy‐resistant and has a poor prognosis. ICIs might be useful treatment options for patients with bladder pleomorphic giant cell carcinoma.

## Introduction

Bladder pleomorphic giant cell carcinoma is a subtype of infiltrating UC described as UC with giant cell carcinoma in the 2016 WHO classification.^1^ It has been reported as a subtype with high malignancy and poor prognosis. It is uncommon, with 23 cases reported to date; among them, 14 cases were diagnosed as muscle‐invasive tumors, of which 10 patients died within 1.5 years after the initial diagnosis.^2^, ^3^, ^4^ Because of its rarity, there are few systemic treatment reports for bladder pleomorphic giant cell carcinoma.

The development of polypoid giant cancer cells is one of the cancer survival strategies induced by the hostile environment and hypoxia.^5^ Such giant cell cancers are dormant for several weeks and have not been regarded crucial as “senescent cells” in the past. However, these cancers survive lethal doses of treatment, produce treatment‐resistant diploid tumor cells, and have metastatic potential.^6^ These cancers take the form of pleomorphic giant cell carcinoma and osteoclast‐like giant cell carcinoma in clinical practice.^6^, ^7^

Pembrolizumab is an ICI that targets the programmed cell death 1 receptor and is approved for advanced‐stage UC previously treated with platinum chemotherapy.^8^ However, evidence for bladder pleomorphic giant cell carcinoma response to ICI drugs, such as pembrolizumab, is lacking.

We report a case of a patient with metastatic bladder pleomorphic giant cell carcinoma who survived for 35 months postoperatively with ICI treatment.

## Case presentation

**Fig. 1:**
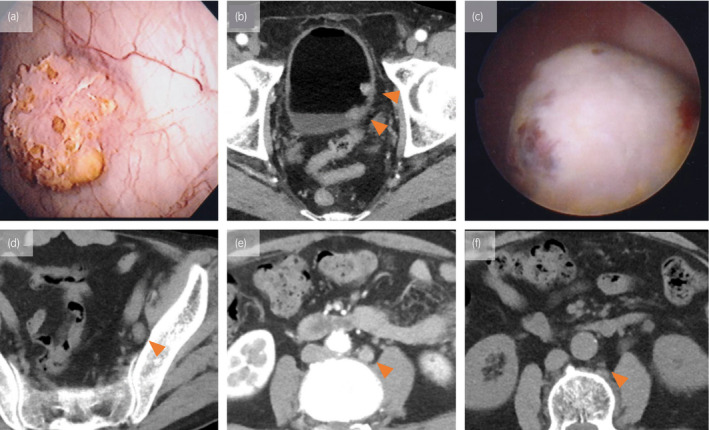
(a) Cystoscopy revealing a papillary tumor at the patient’s first visit. (b) CECT revealing multiple bladder tumors at the patient’s first visit (arrowhead). (c) Cystoscopy at the TURBT after BCG revealing a nodular tumor. (d) CECT revealing the closed left pelvic lymph node metastasis 17 months after the patient's first visit (arrowhead). (e) CECT revealing para‐aortic lymph node metastasis (1.6 × 1.1 cm) 23 months after the first visit (arrowhead). (f) CECT revealing complete remission of para‐aortic lymph node metastasis (1.3 × 1.1 cm) 44 months after the first visit (arrowhead)

**Fig. 2:**
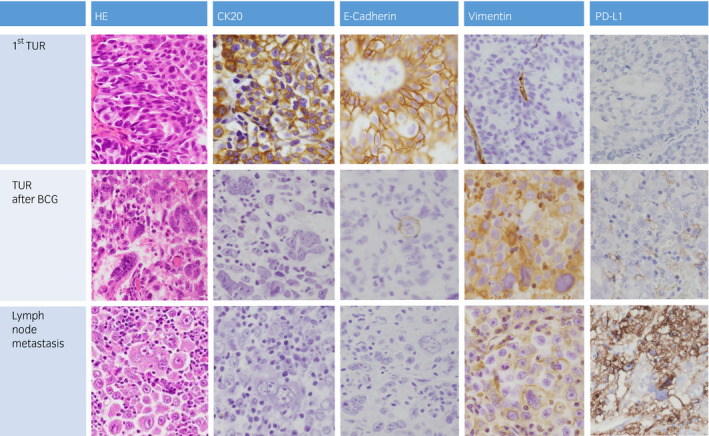
Comparison of conventional hematoxylin‐eosin and immunohistochemical staining of CK20, E‐cadherin, vimentin, and PD‐L1 in paraffin‐embedded tumor tissues among the first TURBT, TURBT after BCG, and left pelvic lymph node metastasis. CK20 and E‐cadherin are strongly positive in differentiated UC cells in the first TURBT and almost negative in undifferentiated carcinoma cells, including mono‐ and multinucleated giant cells, in the TURBT after BCG and left pelvic lymph node metastasis. In contrast, vimentin and PD‐L1 are positive in the TURBT after BCG and left pelvic lymph node metastasis and negative in the first TURBT. PD‐L1 is strongly positive in the pelvic lymph node metastasis. (Brown, immunostaining; purple, counterstain)

A 72‐year‐old man was referred with gross hematuria. He had been smoking for 40 years. Cystoscopy revealed multiple papillary bladder tumors. Therefore, TURBT was performed 1 month after the first visit (Fig. 1a,b). The pathological examination revealed UC pTa of tumor–node–metastasis staging system of the American Joint Committee on Cancer,^9^ WHO grade 3. Therefore, weekly BCG intravesical instillation therapy was administered for 7 weeks. Gross hematuria and nodular tumor were noted 13 months after the first visit (Fig. 1c). The TURBT after BCG was performed 14 months after the first visit. The pathological examination revealed UC stage pT2, WHO Grade 3. Two neoadjuvant chemotherapy courses (gemcitabine/cisplatin) were administered. However, this resulted in the emergence of left pelvic lymph node metastasis (Fig. 1d). Nineteen months later, the patient underwent total cystectomy and pelvic lymphadenectomy. Pathological examination revealed invasive UC with giant cell components pT2N1MX (Fig. 2). Twenty‐three months after the first visit, computed tomography revealed para‐aortic lymph node metastasis (Fig. 1e). Therefore, three gemcitabine/carboplatin combination therapy courses were administered because the patient manifested a slight decrease in renal function after cystectomy. Twenty‐eight months later, pembrolizumab was administered due to para‐aortic lymph node enlargement. Twelve pembrolizumab courses resulted in complete remission (Fig. 1f). Sixteen months after initiation, pembrolizumab was discontinued after 19 courses. The patient had no evidence of disease 30 months after the initiation of pembrolizumab.

## Pathological features

The histopathological examination of the first TURBT revealed a papillary type of noninvasive UC (pTa and WHO grade 3) with tumor cells strongly positive for epithelial markers CAM5.2, CK20, CK7, and E‐cadherin and negative for vimentin. In contrast, the histopathology of the TURBT after BCG showed invasive UC of pT2 and WHO grade 3 in which carcinoma cells became undifferentiated with frequent occurrence of mono‐ and multinucleated pleomorphic giant cells. The morphology of multinucleated giant cells resembled lung pleomorphic giant cell carcinoma, and the cells were CD68 and β‐HCG negative. No spindle cell component was noted in the specimen. The carcinoma cells were immunohistochemically negative for epithelial markers and positive for vimentin and PD‐L1 (Fig. 2). Carcinoma cells of the bladder wall and left pelvic lymph nodes in the radical cystectomy showed almost similar histopathology and immunochemical findings in the TURBT after BCG. However, PD‐L1 immunostaining of the pelvic lymph nodes was strongly positive (Table 1).

**Table 1:** Summary of immunohistochemical stainings

	1st TURBT	TURBT after BCG	Radical cystectomy	
			Bladder	Lymph node
CAM5.2	3+	3+	2+	2+
CK7	3+	0	0	0
CK20	3+	0	0	0
E‐cadherin	3+	0	0	0
Vimentin	0	2+	1+	1+
PD‐L1	0	1	–	3+
p53	3+	3+	3+	3+

3+ strongly positive, 2+ moderately positive, 1+ weakly positive, 0 negative.

## Discussion

Lopez *et al.* defined bladder polymorphic giant cell carcinoma as “a rare variant of UC with histologic appearance similar to giant cell carcinoma of the lung.”^2^, ^3^ Osteoclast‐like giant cell carcinoma, syncytiotrophoblastic giant cell carcinoma, sarcomatoid carcinoma, and pleomorphic giant cell carcinoma are bladder cancer subtypes that exhibit the morphology of giant cell carcinoma.^1^ In this case, osteoclast‐like giant cell carcinoma was ruled out because osteoclast mesenchymal markers such as CD68 were negative. Syncytiotrophoblastic giant cells were ruled out as β‐HCG was negative. No spindle cell component was found in the tissue, so sarcomatoid bladder carcinoma was ruled out. Therefore, we finally diagnosed this case as bladder pleomorphic giant cell carcinoma.

In the present case, high PD‐L1 expression was observed in the tumor after neoadjuvant chemotherapy. Cisplatin and carboplatin‐based chemotherapy increase PD‐L1 expression by tumor cells.^10^ Although high PD‐L1 expression is a poor prognostic factor for chemotherapy, it is an effective target for pembrolizumab.^11^ High PD‐L1 expression after neoadjuvant chemotherapy may have led to complete remission in the present case. We believe that bladder pleomorphic giant cell carcinoma is a simple high chemoresistant type of UC.^7^ Because of a different mechanism from chemotherapy, pembrolizumab may be effective in treating bladder pleomorphic giant cell carcinoma as with the treatment of UC.

We should have opted for pembrolizumab over carboplatin for systemic treatment of postoperative recurrence in this case after cystectomy. Both giant cell development and high PD‐L1 expression may lead to chemotherapy resistance and poor prognosis. Moreover, immediate total cystectomy may have been a treatment option for bladder pleomorphic giant cell carcinoma because of this chemoresistance. Kimura *et al.* reported a successful case of muscle‐invasive bladder pleomorphic giant cell carcinoma after neoadjuvant chemotherapy.^4^ However, in this case, the effect of neoadjuvant chemotherapy was limited, resulting in pelvic lymph node metastasis. Future studies are required regarding systemic treatment for bladder pleomorphic giant cell carcinoma.

According to the European Association of Urology guidelines, our case is at high risk of recurrence.^12^ Therefore, we initiated BCG intravesical instillation therapy after the first TURBT. Tumors in TURBT after BCG were giant cell carcinomas that were negative for most epithelial markers and positive for some mesenchymal markers (Fig. 2, Table 1). BCG intravesical instillation therapy causes inflammation and oxidative stress in the bladder.^13^ These might cause giant cell development and epithelial–mesenchymal transition in the remaining bladder tumor after the first TURBT.^14^, ^15^ However, we cannot rule out the possibility that a new giant cell carcinoma developed in the bladder after BCG intravesical instillation therapy. Future studies should investigate the relationship between BCG intravesical instillation therapy and giant cell cancer development.

## Conclusion

This is the first reported successful case of pembrolizumab treatment in metastatic bladder pleomorphic giant cell carcinoma. Based on this observation, ICIs appear to be useful for treating bladder pleomorphic giant cell carcinoma; however, further studies are required to validate this observation.

## Conflict of interest

The authors declare no conflict of interest.
